# Post-miscarriage Complement-Mediated Thrombotic Microangiopathy in a 27-Year-Old Woman: A Case Highlighting Diagnostic and Therapeutic Gaps in Brazil

**DOI:** 10.7759/cureus.99685

**Published:** 2025-12-20

**Authors:** Francisco Brenon O Torres, Leticia O Souza Santos, Nivia L Chagas Pereira, Lucas S Uchôa Xavier Rodrigues, Juliana L Santana de Oliveira

**Affiliations:** 1 Intensive Care Unit, Doctor Nise da Silveira Women's Hospital, Maceió, BRA; 2 Intensive Care Unit, Unimed Maceió Hospital, Maceió, BRA; 3 Medicine, State University of Health Sciences of Alagoas, Maceió, BRA; 4 Intensive Care Unit, Campinas Maternity Hospital, Campinas, BRA; 5 Emergency, Hospital Memorial Arthur Ramos, Maceió, BRA

**Keywords:** acute kidney injury, atypical hemolytic uremic syndrome (ahus), complement system proteins, disseminated intravascular coagulation, pregnancy complications, spontaneous abortion, thrombotic microangiopathies

## Abstract

Thrombotic microangiopathies (TMAs) comprise a group of severe diseases characterized by microangiopathic hemolytic anemia, thrombocytopenia, and organ dysfunction. Differential diagnosis among subtypes such as complement-mediated TMA (atypical hemolytic uremic syndrome (aHUS)), thrombotic thrombocytopenic purpura (TTP), and typical hemolytic uremic syndrome (HUS) is particularly challenging in settings with limited diagnostic and therapeutic resources. We report the case of a 27-year-old patient, G2P0A1, admitted to a private Brazilian hospital at 19 weeks of gestation with a pregnancy complicated by anhydramnios and spontaneous abortion, followed by difficult-to-control bleeding and rapid clinical deterioration. After the abortion and uterine curettage, the patient developed the classic triad of thrombotic microangiopathy in addition to high fever, hypovolemic shock, severe metabolic acidosis, acute kidney injury with anuria requiring hemodialysis, significant coagulopathy, and marked elevation of transaminases. The diagnostic workup was hampered by the unavailability of key tests, such as A Disintegrin And Metalloprotease with ThromboSpondin type 1 motif, member 13 (ADAMTS13) activity and complement genetic testing. Nevertheless, the combination of (1) TMA triad, (2) reduced complement level (C3), (3) low risk of TTP according to the PLASMIC score and absence of need for plasmapheresis, (4) lack of preceding diarrhea suggestive of typical HUS, (5) negative cultures and no specific response to antimicrobial therapy, and (6) a clinical course incompatible with Hemolysis, Elevated Liver enzymes, and Low Platelets (HELLP) syndrome supported the diagnosis of complement-mediated TMA, likely triggered by obstetric complications and associated with disseminated intravascular coagulation (DIC). Twelve days after the initial event, repeat ultrasound revealed retained products of conception, and a second curettage was performed, followed by progressive clinical improvement. Despite this, the patient evolved to permanent renal failure with dialysis dependence due to lack of access to targeted therapies such as eculizumab, which could potentially have modified the prognosis. This case illustrates how, even in private hospitals, the limited availability of diagnostic tests and specific therapies compromises adequate differential diagnosis of TMAs and influences critical outcomes. It highlights the significant impact of structural barriers on the management of rare and complex diseases such as aHUS in Brazil.

## Introduction

Thrombotic microangiopathies (TMAs) represent a heterogeneous group of disorders characterized by microangiopathic hemolytic anemia, thrombocytopenia, and target-organ damage, most frequently involving the kidneys and central nervous system [1]. Among TMAs, complement-mediated TMA, also known as atypical hemolytic uremic syndrome (aHUS), is particularly challenging due to its complex pathophysiology, rarity, and the need for precise differential diagnosis from other TMAs such as thrombotic thrombocytopenic purpura (TTP), Hemolysis, Elevated Liver enzymes, and Low Platelets (HELLP) syndrome, and disseminated intravascular coagulation (DIC) [2-4]. Notably, a substantial proportion of pregnancies complicated by aHUS occur in the setting of severe obstetric complications, including preeclampsia, HELLP syndrome, postpartum hemorrhage, and pregnancy loss, which further complicate timely diagnosis and management [5,6].

In Brazil, the diagnosis and management of TMAs are further complicated by structural barriers to advanced diagnostic tests and complement-targeted therapies. In the public health system, A Disintegrin And Metalloprotease with ThromboSpondin type 1 motif, member 13 (ADAMTS13) activity assays and complement inhibitors (eculizumab and ravulizumab), humanized monoclonal antibodies that block complement component C5 and currently represent the standard etiologic treatment for complement-mediated TMAs, including atypical HUS, are generally not available in routine practice. In practice, ADAMTS13 activity is performed only in a few tertiary public referral or university centers and central reference laboratories and is not routinely accessible to most public or private hospitals. Although, in theory, private hospitals may refer patients or samples to these centers, geographical distance, administrative barriers, and the need for rapid decision-making in acutely ill TMA patients often limit the feasibility of such referral pathways in the acute setting. Advanced diagnostic tools for TMA include ADAMTS13 activity assays, which are used to document severe ADAMTS13 deficiency typical of immune TTP, and complement testing (C3 and C4 levels and, when available, functional assays), which can support the diagnosis of complement-mediated TMAs such as aHUS [3,5]. In the private sector, these resources are formally accessible but often depend on outsourced reference laboratories and complex logistical arrangements, leading to delays that are incompatible with acute decision-making. These limitations, particularly in Northeastern Brazil, may prolong or even preclude the diagnostic journey for patients with suspected TMA, with a potential negative impact on prognosis.

This report describes a complex clinical case of complement-mediated TMA associated with obstetric complications in a private Brazilian hospital, highlighting the impact of unavailability of essential diagnostic tests and specific treatments on differential diagnosis, clinical management, and patient outcomes. The case also illustrates how the overlap of severe conditions further complicates diagnostic definition in resource-limited settings.

## Case presentation

**Figure 1:**
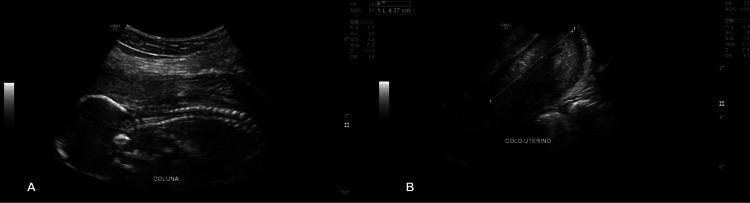
Obstetric transvaginal ultrasound at approximately 19 weeks of gestation showing absolute oligohydramnios (A) and a cervical length of 4.3 cm with a slightly opened internal os (B). FR, frame rate; AO, acoustic output; CHI, coded harmonic imaging; Frq, transducer frequency; Gn, overall gain; S/A, image filtering setting specific to the ultrasound system; D, image depth; DR, dynamic range; os, cervical os.

A 27-year-old woman, G2P0A1, with a history of previous late miscarriage of unknown cause, was admitted to a private hospital at 19 weeks of gestation with mild vaginal bleeding, abdominal cramping, and fluid loss, consistent with preterm premature rupture of membranes (PPROM). Transvaginal ultrasound revealed anhydramnios and a dilated cervical os (Figure 1). As there were no clinical signs of active labor at admission, she was initially managed expectantly with close monitoring and prophylactic intravenous ampicillin 2 g every six hours, without tocolysis. Antenatal corticosteroids were not administered because the gestational age was 19 weeks, below the local threshold of fetal viability. Despite these measures, she progressed to spontaneous abortion within a few hours and underwent uterine curettage, which was complicated by difficult-to-control bleeding. She was therefore transferred to the intensive care unit (ICU) for hemodynamic monitoring and intensive care.

**Figure 2:**
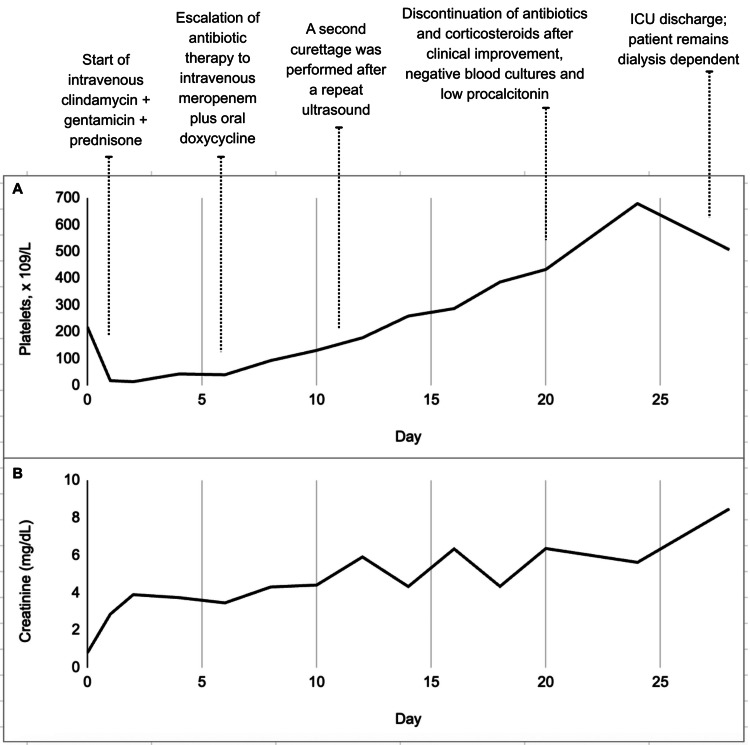
Temporal evolution of hematologic and renal parameters. (A) Platelet count (×10⁹/L) from day 0 (hospital admission) to day 28, showing an initial nadir followed by progressive hematologic recovery. (B) Serum creatinine (mg/dL) over the same period, demonstrating persistent and progressive renal dysfunction despite hematologic improvement. The upper timeline indicates the main therapeutic interventions, including initiation and escalation of antibiotic therapy, second curettage, discontinuation of antibiotics and corticosteroids, and ICU discharge.

Upon ICU admission, her condition rapidly worsened, with progression to shock and severe metabolic acidosis. Orotracheal intubation and vasoactive support were required for hemodynamic stabilization. Initial laboratory tests were already alarming, revealing the triad of thrombotic microangiopathy: severe thrombocytopenia (18,000 platelets/mm³), microangiopathic hemolytic anemia (hemoglobin: 7.4 g/dL, lactate dehydrogenase (LDH): > 5,000 U/L, and the presence of schistocytes), and acute kidney injury Kidney Disease: Improving Global Outcomes (KDIGO) stage 3 (creatinine: 2.86 mg/dL followed by anuria), requiring immediate hemodialysis. In addition, she presented marked transaminase elevation (aspartate aminotransferase (AST) and alanine aminotransferase (ALT): 1,487 U/L and 773 U/L, respectively), coagulopathy (international normalized ratio (INR): 2.67 and fibrinogen: 159 mg/dL in the setting of ongoing bleeding), and signs suggestive of infection (fever, leukocytosis, and a urine culture positive for *Escherichia coli*), which created a major challenge in differentiating between sepsis, DIC, and TMA. Serial hematologic, renal, and biochemical parameters during hospitalization are summarized in Table 1, and the temporal evolution of platelet count and serum creatinine, along with the timing of major therapeutic interventions, is depicted in Figure 2.

**Table 1:** Serial hematologic, renal, and biochemical parameters during hospital stay. hCG: human chorionic gonadotropin; INR, international normalized ratio; LDH, lactate dehydrogenase; Hb, hemoglobin; CRP, C-reactive protein; AST, aspartate aminotransferase; ALT, alanine aminotransferase; RV, reference values. Day = days after hospital admission. Blank cells indicate that the parameter was not measured on those days.

Parameter	Day													
	0	1	2	4	6	8	10	12	14	16	18	20	24	28
β-hCG (mIU/mL)		4,935		1,141					257					
Hb (g/dL)	10.7	7.4	8.2	7.3	7.7	7.1	7.9	6.8	8	7.7	7.7	7.7	8.3	8.2
Reticulocytes			12%									2.5%		
Haptoglobin			18								157			
Platelets, x 10^9^/L	218	18	14	43	40	93	131	178	259	287	386	433	679	507
Leukocytes, x 10^9^/L	2.2	36.9	69.6	70	100.3	92.4	89.9	59.4	43.6	34.4	25.8	18.1	6.1	8.8
Creatinine (mg/dL)	0.8	2.86	3.9	3.74	3.46	4.31	4.41	5.91	4.33	6.34	4.34	6.36	5.62	8.47
Urea (mg/dL)	41	69	45	40	65	148		218	143	194	107	163	118	71
LDH (U/L)		5064							891					
Total bilirubin (mg/dL)		1.91		5.93							2.54			1.04
AST (U/L)		1,487		325							30			
ALT (U/L)		773									10			
CRP (mg/L)		501	663		380	161	179	80.3	85			57.8		16.2
Fibrinogen, mg/dL (RV: 180-420)		159									570			
INR		2.67	3.69	1.19	1.2							1.09		
D-dimer (µg/mL) (RV: 0-0.5)		>10									0.4			
Procalcitonin (ng/mL)			1.68						0.76			0.46		
Lactate (mg/dL)		286		33.7										11.3
Potassium (mmol/L)		5	4	3.7	3.5	2.7	4.1	3.9	4.1	4.3	4.4	4.8	5.5	4.5
Sodium (mEq/L)		138	135	137	137	136	135	136	135	131	130	127	124	

Urinalysis and urinary sediment were performed to further characterize the acute kidney injury. The urinary sediment was consistent with acute tubular injury, without dysmorphic red blood cells, red blood cell casts, or other features suggestive of primary glomerulonephritis. An immunologic workup was obtained to investigate underlying systemic autoimmune disease. Antinuclear antibodies and antiphospholipid antibodies were negative, providing no evidence for systemic lupus erythematosus or antiphospholipid syndrome. Serum vitamin B12 levels were within the normal range, making vitamin B12-related metabolic HUS unlikely. Plasma homocysteine ​​levels were not available in our setting and are recognized as a limitation of the diagnostic workup. The immunologic and metabolic workup for thrombotic microangiopathy, including complement levels and tests for systemic autoimmune disease and vitamin B12-related HUS, is shown in Table 2.

**Table 2:** Immunologic and metabolic workup for thrombotic microangiopathy. DAT, direct antiglobulin test; ANA, antinuclear antibody. Day = days after hospital admission. Blank cells indicate that the parameter was not measured on those days.

Parameter	Day													
	0	1	2	4	6	8	10	12	14	16	18	20	24	28
Vitamin B12 (pg/mL)					254									
Complement C3 (g/L)				0.61										
DAT	Negative													
ANA	Negative													
Anticardiolipin IgG/IgM	Negative													
Anti-β2 glycoprotein I IgG/IgM	Negative													
Lupus anticoagulant	Negative													

The diagnostic workup was complex and hindered by the absence of specific diagnostic resources, and intensive care was required for four weeks. On ICU admission, hemoglobin was 7.4 g/dL, associated with severe thrombocytopenia, and the patient received multiple packed red blood cell and platelet transfusions. Broad-spectrum antimicrobial therapy with intravenous clindamycin 600 mg every six hours plus gentamicin 6 mg/kg/day was initiated to cover the possibility of septic shock. Because TTP and HELLP syndrome were strongly suspected, corticosteroid therapy with prednisone 1 mg/kg/day was started, and a hematology consultation was obtained. The hematology team confirmed microangiopathic hemolytic anemia (negative Coombs test, presence of erythroblasts and schistocytes, anemia, and severe thrombocytopenia).

Complement levels (C3 and C4) were measured and revealed reduced C3, a crucial finding that supported the suspicion of a complement-mediated TMA. TTP was considered unlikely given a PLASMIC score [7] of 4 (low risk) and the favorable clinical evolution without plasma exchange. Shiga toxin testing for Shiga toxin-producing *Escherichia* *coli*-associated hemolytic uremic syndrome (STEC-HUS) (stool PCR or culture) was not available in our institution, and this etiology could therefore not be definitively excluded. However, the absence of a diarrheal prodrome, the local epidemiological background, and the presence of hypocomplementemia made pregnancy-associated aHUS presenting with DIC the most likely diagnosis. It is also important to note that additional investigations, including bone marrow aspiration, immunophenotyping, and karyotyping, were performed to exclude hematologic malignancies and confirmed a reactive process.

**Figure 3:**
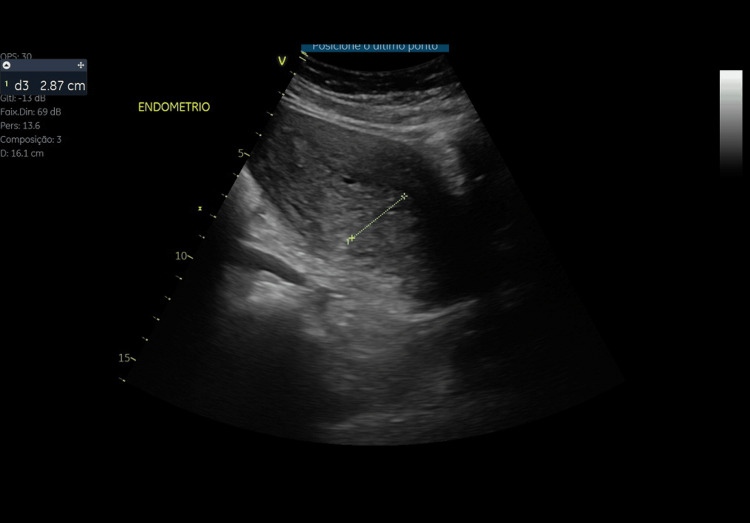
Transabdominal and transvaginal pelvic ultrasound demonstrating heterogeneous material within the endometrial cavity, consistent with retained products of conception.

Despite intensive supportive care, recovery was slow. A repeat pelvic ultrasound performed 12 days after admission revealed heterogeneous material within the endometrial cavity, consistent with retained products of conception (Figure 3). The patient underwent a second curettage, after which she showed progressive clinical improvement, hemodynamic stabilization, and hematologic recovery over the following weeks. Antimicrobial therapy, which had been initiated on ICU admission with intravenous clindamycin plus gentamicin, was escalated on day 6 of ICU stay to intravenous meropenem 1 g every eight hours and oral doxycycline 100 mg every 12 hours, due to persistent clinical instability and the identification of hydrosalpinx on pelvic ultrasound. The full course of antibiotics lasted 20 days and was discontinued on day 20 of ICU stay, when multiple sets of blood cultures remained negative and procalcitonin was <0.5 ng/mL, making sepsis unlikely. Corticosteroid therapy was progressively tapered and completely withdrawn on the same day, in parallel with sustained clinical and laboratory improvement.

After one month of hospitalization, the patient was discharged from intensive care. However, due to severe kidney injury, she remained dialysis dependent. At that time, a kidney biopsy was performed to further evaluate the cause and chronicity of kidney damage. Histology showed chronic eosinophil-rich tubulointerstitial nephritis in activity, with moderate interstitial fibrosis and tubular atrophy and a high proportion of globally sclerotic glomeruli, while immunofluorescence was negative for immunoglobulins, complement, and fibrinogen. This case illustrates that survival was achieved through intensive supportive care, but also highlights the unfavorable renal outcome due to the lack of access to targeted therapies such as eculizumab.

## Discussion

This case report illustrates the complexity of the differential diagnosis of thrombotic microangiopathies (TMAs), especially in a severe clinical setting, where the initial presentation may mimic and be associated with other conditions requiring distinct clinical management. The patient in question, with a history of previous late miscarriage of unknown cause, developed shock, multiple organ failure, microangiopathic hemolytic anemia, and severe thrombocytopenia after a new late spontaneous abortion and curettage. As highlighted in recent series, a substantial proportion of pregnancies complicated by aHUS occur in the setting of severe obstetric complications such as preeclampsia, HELLP syndrome, postpartum hemorrhage, and pregnancy loss, which may act as potent triggers of complement activation in genetically susceptible women [5,8]. In our case, the combination of late miscarriage, postpartum hemorrhage, DIC, and severe infection likely created a “perfect storm” for the development of pregnancy-associated aHUS.

The early course, characterized by coagulopathy and bleeding, raised suspicion of disseminated intravascular coagulation [4], whereas marked leukocytosis and a urine culture positive for *E. coli* initially directed the investigation toward septic shock secondary to urinary tract sepsis [1]. After sepsis was ruled out by persistently negative cultures, and despite the lack of genetic tests to demonstrate complement system dysregulation, the presence of the TMA triad allied with C3 hypocomplementemia strongly supported a diagnosis of complement-mediated TMA [5]. Furthermore, although aHUS may be associated with mild coagulation abnormalities, the presence of significant coagulopathy and difficult-to-control bleeding suggests an overlap with DIC, likely triggered by obstetric complications [6,8-14]. These clinical and laboratory features, together with the marked dynamic changes in platelet count, hemoglobin, creatinine, LDH, and liver enzymes, are consistent with the evolution of a severe TMA in the context of DIC (Table 1).

Differentiating between the possible causes of TMA proved to be a significant challenge. Exclusion of TTP was based primarily on clinical improvement without plasmapheresis and the low-risk PLASMIC score (4 points), given the unavailability of ADAMTS13 activity testing. Shiga toxin testing for STEC-HUS (stool PCR or culture) was not available in our institution, and this etiology could therefore not be definitively excluded. However, the absence of a diarrheal prodrome, the local epidemiological background, and the presence of hypocomplementemia made pregnancy-associated aHUS presenting with DIC the most likely diagnosis. In addition, HELLP syndrome was deemed improbable due to the early gestational age and predominance of renal impairment, supporting the hypothesis of aHUS [1]. Close collaboration with the hematology team and the performance of bone marrow aspiration, immunophenotyping, and karyotyping were essential to exclude hematologic malignancies as the cause of leukocytosis and hematologic abnormalities, confirming a reactive process. In parallel, the immunologic and metabolic workup helped to exclude autoimmune and vitamin B12-related forms of TMA: antinuclear antibodies and antiphospholipid antibodies were negative, and serum vitamin B12 levels were within the normal range, whereas plasma homocysteine levels were not available (Table 2).

Renal biopsy plays an important role in excluding alternative causes of kidney injury in patients with TMA, even though kidney morphology often does not reveal the specific etiology of the microangiopathy. In our patient, kidney biopsy was not performed during the ICU stay but was carried out after intensive care discharge. Histology showed chronic eosinophil-rich tubulointerstitial nephritis in activity, with moderate interstitial fibrosis and tubular atrophy, a high proportion of globally sclerotic glomeruli and negative immunofluorescence for immunoglobulins, complement, and fibrinogen. We interpreted these findings as reflecting advanced chronic scarring after severe ischemic and microangiopathic injury associated with the obstetric catastrophe, DIC, and shock, superimposed on an eosinophil-rich tubulointerstitial process compatible with concomitant interstitial nephritis. This pattern is consistent with the chronic stage of kidney damage and does not exclude the occurrence of complement-mediated TMA associated with pregnancy at the time of the acute event.

From a pathophysiological standpoint, assessment of terminal complement activation through measurement of soluble membrane attack complex (MAC, C5b-9) has emerged as a potentially useful and relatively accessible biomarker of complement cascade activation, particularly in settings where genetic or advanced functional complement tests are not available [15]. Unfortunately, MAC (C5b-9) levels were not measured in our patient due to logistical difficulties in sending samples to an external reference laboratory, and we recognize this as an additional limitation of the present report.

Our observations are consistent with recent case reports and series describing pregnancy-associated complement-mediated HUS precipitated by severe obstetric complications, including postpartum hemorrhage, HELLP syndrome and sepsis, in which delayed recognition of TMA and restricted access to C5 inhibitors were associated with poor renal outcomes and a high rate of dialysis dependence [5,8]. Conversely, early initiation of eculizumab or ravulizumab has been associated with more favorable renal recovery in similar scenarios [3,5,8].

This case underlines the diagnostic and therapeutic barriers faced in Brazil, even within the private healthcare system. Delayed access to diagnostic tests and difficulty obtaining targeted therapies prolong investigation and length of hospitalization and limit specific treatment options. Although the patient survived due to intensive supportive care, the outcome was unfavorable, with irreversible renal failure and permanent dialysis dependence, an outcome that might have been avoided with earlier diagnosis and timely access to complement-modulating therapies [5,8]. This scenario is likely even more critical in public hospitals, where limited availability of basic tests such as fibrinogen, LDH, and haptoglobin is common, making the differential diagnosis of TMAs an almost insurmountable challenge.

## Conclusions

This case of complement-mediated TMA in a patient following late abortion illustrates the extreme diagnostic and therapeutic difficulties in resource-limited settings, particularly when overlapping with other severe conditions. Clinical suspicion, supported by the exclusion of other etiologies in the absence of gold-standard tests, was crucial in guiding management. However, the unavailability of specific diagnostic assays and the lack of access to complement inhibitors contributed to an unfavorable outcome, culminating in irreversible kidney injury. From a preventive standpoint, this case underscores the importance of early recognition of the TMA triad in pregnancy and the postpartum period, systematic evaluation for TTP, STEC-HUS, and HELLP syndrome, and early referral to tertiary centers where complement testing and C5 inhibitors may be available, as these measures may reduce the risk of irreversible kidney damage. In survivors who progress to chronic kidney disease or dialysis dependence, maintaining quality of life requires structured long-term nephrology follow-up, optimization of dialysis, cardiovascular risk factor control, and psychosocial support, with timely evaluation for kidney transplantation when appropriate. Patients and their families should be counselled about the nature of complement-mediated TMA, the risk of recurrence, particularly in future pregnancies or other complement-activating conditions, and the need to seek early medical attention if warning symptoms such as fatigue, edema, reduced urine output, or neurological changes occur, as well as about available strategies to support functional recovery and social reintegration.
